# Molecular (PCR-DGGE) versus morphological approach: analysis of taxonomic composition of potentially toxic cyanobacteria in freshwater lakes

**DOI:** 10.1186/2046-9063-10-2

**Published:** 2014-02-12

**Authors:** Aleksandra Bukowska, Aleksandra Bielczyńska, Anna Karnkowska, Ryszard J Chróst, Iwona Jasser

**Affiliations:** 1 Microbial Ecology Department, Faculty of Biology, Institute of Botany, University of Warsaw, ul. Miecznikowa 1, Warsaw, Poland; 2 Department of Systematics and Plant Geography, Faculty of Biology, Institute of Botany, University of Warsaw, Aleje Ujazdowskie 4, Warsaw, Poland

**Keywords:** Cyanobacteria, DGGE, ITS, *mcy* genes, Microcystins, Microscopic analysis

## Abstract

**Background:**

The microscopic Utermöhl method is commonly used for the recognition of the presence and taxonomic composition of potentially toxic cyanobacteria and is especially useful for monitoring reservoirs used as drinking water, recreation and fishery resources. However, this method is time-consuming and does not allow potentially toxic and nontoxic cyanobacterial strains to be distinguished. We have developed a method based on denaturing gradient gel electrophoresis (DGGE) of the marker gene ITS and the *mcy*-gene cluster, and DNA sequencing. We have attempted to calibrate the DGGE-method with a microscopic procedure, using water samples taken in 2011 from four lakes of the Great Mazurian Lakes system.

**Results:**

Results showed that the classic microscopic method was much more precise and allowed the classification of the majority of cyanobacterial taxa to the species or genus. Using the molecular approach, most of the sequences could only be assigned to a genus or family. The results of DGGE and microscopic analyses overlapped in the detection of the filamentous cyanobacteria. For coccoid cyanobacteria, we only found two taxa using the molecular method, which represented 17% of the total taxa identified using microscopic observations. The DGGE method allowed the identification of two genera of cyanobacteria (*Planktothrix* and *Microcystis*) in the studied samples, which have the potential ability to produce toxins from the microcystins group.

**Conclusions:**

The results confirmed that the molecular approach is useful for the rapid detection and taxonomic distinction of potentially toxic cyanobacteria in lake-water samples, also in very diverse cyanobacterial communities. Such rapid detection is unattainable by other methods. However, with still limited nucleotide sequences deposited in the public databases, this method is currently not sufficient to evaluate the entire taxonomic composition of cyanobacteria in lakes.

## Background

Cyanobacteria are a group of prokaryotic autotrophic microorganisms that commonly occur in both marine and freshwater environments. The massive appearance of cyanobacteria creates a serious threat to aquatic ecosystems. In the summer period, cyanobacteria (commonly called blue-green algae) often become the dominant component of the phytoplankton community in eutrophicated lake ecosystems [1]. Their blooms, which are promoted by the large amounts of phosphorus, high temperature, a decrease in the total nitrogen:total phosphorus (TN:TP) ratio and lack of, or slow water hydraulic flow, can lead to a decrease in aquatic biota diversity, increased turbidity and oxygen deficit of the water. Furthermore, the increased concentration of toxins produced by cyanobacteria is dangerous for aquatic biota and humans, especially when present in drinking water reservoirs, or in water bodies used for recreational and fishery purposes [2].

Many species of cyanobacteria can produce toxins such as hepatotoxins, neurotoxins and dermatotoxins. Freshwater cyanobacteria that belong to the genera *Microcystis*, *Anabaena*, *Dolichospermum*, *Planktothrix* and *Nostoc*, produce microcystins (hepatotoxins), which are the most common cyanobacterial toxins in freshwaters [2]. These are cyclic heptapeptides; secondary metabolites that are synthesized non-ribosomally. At the molecular level, the *mcy*-gene cluster, which encodes the enzymes that synthesize toxins, is responsible for the production of microcystins [3]. The aquatic community of cyanobacteria can comprise both toxic and non-toxic strains but only the presence of all 7 genes that are involved in microcystins synthesis (*mcy*A, *mcy*B, *mcy*C, *mcy*D, *mcy*E, *mcy*G, and *mcy*J), allows the strains to produce toxins, assuming that there are no mutations in the cluster, in which case microcystins might still not be produced [4].

Analyses of cyanobacterial diversity provide information concerning the condition of a reservoir, because the taxonomic composition and proportions of different cyanobacterial taxa in water change with increasing trophic status. Filamentous forms mostly dominate in lakes with a higher eutrophic or hypertrophic level, whereas in oligotrophic and mesotrophic waters, much lower densities of these cyanobacterial forms occur [1].

The classic method for examination of the cyanobacteria taxonomic composition, the Utermöhl method [5], is based on the use of an inverted microscope analysis of water samples. This method is accurate and informative, but has some limitations. One disadvantage is that it is time-consuming and leads to a high frequency of errors, due to the fact that many taxa appear very similar. It also requires a very high degree of taxonomic expertise from researchers performing it. However, it does allow the classification of many taxa to the species level, which is often unattainable by other methods. Because of their very small cell-size, the presence of picocyanobacteria cannot be determined by the Utermöhl method, thus for this purpose, epifluorescence microscopy is usually used.

The Utermöhl method also only allows the identification of cyanobacteria that occur in lake water samples and does not provide information concerning their possible toxicity, because it is impossible to distinguish potentially toxic and non-toxic strains based on phenotypic traits. The ability to identify genotypes that possess toxicity genes might allow the prediction of whether cyanobacterial blooms are toxic when they occur, which is particularly important in the case of commercial reservoirs.

Molecular methods, which are now widely used in environmental studies, not only allow the evaluation of the taxonomic composition of cyanobacteria, but also the identification of groups of cyanobacteria that contain the *mcy*-gene cluster in their genomes.

Denaturing gradient gel electrophoresis (DGGE) is a technique for separating amplified double-stranded DNA fragments of identical length, which differ only in nucleotide composition, using the differences in the stability of AT and GC pairs [6]. DNA fragments for this method are specifically amplified from selected organisms and should be variable enough to be distinguished between different groups. At the same time, as many sequences from different taxa as possible should be published to enable the comparison and identification of sequences [7].

The internal transcribed spacer (ITS) is a fragment located between the 16S and 23S rRNA genes. It has greater degree of sequence heterogeneity than 16S rRNA sequence and thus allows many genera of cyanobacteria to be distinguished [7]. The *mcy*A gene is one of the *mcy*-cluster genes, whose sequence enables the discrimination of different cyanobacterial genera, which can potentially produce microcystins [8].

The advantage of this molecular method is clearly the shorter length of time required compared to microscopic analyses and the ability to verify the presence of toxic strains within detected taxa. However, it is often not possible to generate PCR primers that are completely specific to all targeted taxa. Furthermore, this method does not allow the assessment of the number and biomass of cyanobacteria; only the proportions of different genotypes in each sample can be estimated.

The aim of this study was to compare the two methods of identification of cyanobacteria as well as an attempt to calibrate the classic Utermöhl method and the molecular method, using water samples collected from four lakes of the Great Mazurian Lakes system characterised by a very diverse community of cyanobacteria. The second aim of the study was to check which of the taxa present in studied lakes posses the *mcy*A-genes.

## Results and discussion

The number of taxa detected in each water sample by microscopy was compared with the number of bands obtained from DGGE-ITS analyses (Table 1). The largest number of taxa (29) using the microscopic analyses was detected in water samples from Lake Tałtowisko obtained in August, whereas the smallest number (6) was observed in samples from Lake Bełdany in May. The largest number of bands obtained using DGGE (29) was found in Lake Mikołajskie in July, whereas in the May samples from lakes Tałtowisko and Mamry, no bands were detected. Fewer taxa, of a mean of 3.2 (bands), were found using molecular analyses than using the microscopic method. Table 2 presents comparison of composition of cyanobacterial community obtained by both methods in two exemplary samples collected from lakes Mikołajskie and Mamry in September. It demonstrates differences in the acquired number of taxa as well as the difference in the taxonomic level of identification achieved in each of the methods.

**Table 1:** Comparison of numbers of cyanobacterial taxa in microscopic analyses and numbers of bands obtained from DGGE-ITS analyses

**Month**	**Lake**	**Taxa (microscope)**	**DGGE-ITS bands**
May	Mamry	7	0
	Tałtowisko	9	0
	Mikołajskie	7	6
	Bełdany	6	3
July	Mamry	20	24
	Tałtowisko	27	20
	Mikołajskie	20	29
	Bełdany	23	22
August	Mamry	22	27
	Tałtowisko	29	17
	Mikołajskie	24	16
	Bełdany	25	20
September	Mamry	27	20
	Tałtowisko	20	16
	Mikołajskie	17	17
	Bełdany	20	15

**Table 2:** The entire taxonomic composition of the cyanobacterial community in two selected samples, evaluated using microscopic and molecular methods

**Mikołajskie september**			**Mamry september**		
**Microscope**		**DGGE-ITS**	**Microscope**		**DGGE-ITS**
**taxon**	**(cells/mL)**		**taxon**	**(cells/mL)**	
*Snowella litoralis*	3		*Snowella litoralis*	118	
*Snowella lacustris*	31		*Snowella lacustris*	1	
			*Woronichinia compacta*	1	
			*Woronichinia naegeliana*	2	
*Chroococcus* spp.	<1		*Chroococcus* spp.	13	
			*Aphanothece* spp.	779	
			*Microcystis aeruginosa*	19	*Microcystis*
			*Microcystis flos-aquae*	7	
			*Microcystis smithii*	5	
*Aphanocapsa* spp.	34		*Aphanocapsa* spp.	4599	
*Aphanocapsa planctonica*	4		*Aphanocapsa planctonica*	75	
			*Coelosphaerium kuetzingianum*	2	
*Coelosphaerium minutissimum*	3		*Coelosphaerium minutissimum*	18	
			*Merismopedia punctata*	1	
*Cyanodictyon reticulatum*	2				
			*Cyanodictyon* cf. *planctonicum*	7	
			*Lemmermanniella pallida*	4	
*Synechococcus*-like*/Cyanobium*-like	60042	*Synechococcus*	*Synechococcus*-like/*Cyanobium*-like	76386	*Synechococcus*
*Aphanizomenon gracile*	839	*Aphanizomenon* (?)	*Aphanizomenon gracile*	336	*Aphanizomenon* (?)
*Cuspidothrix issatschenkoi*	20	*Cuspidothrix issatschenkoi*	*Cuspidothrix issatschenkoi*	25	*Cuspidothrix issatschenkoi*
		*Dolichospermum*	*Dolichospermum lemmermannii*	1	*Dolichospermum*
		*Phormidium* (?)			*Phormidium* (?)
*Planktothrix agardhii*	45	*Planktothrix*			*Planktothrix*
*Planktothrix suspensa*					
		*Pseudanabaenaceae*			*Pseudanabaenaceae*
					*Leptolyngbya*
*Limnothrix lauterbornii*	128		*Limnothrix lauterbornii*	154	
*Limnothrix redekei*	692		*Limnothrix redekei*	742	
*Planktolyngbya limnetica*	5455		*Planktolyngbya limnetica*	3293	
*Pseudanabaena catenata*	5963		*Pseudanabaena catenata*	1393	
*Pseudanabaena limnetica*	18172		*Pseudanabaena limnetica*	24635	
			*Pseudanabaena mucicola*	10	
		*Romeria* (?)	*Romeria gracilis*	214	*Romeria* (?)
		unassigned sequences			unassigned sequences
		nonsequenced			nonsequenced

Question marks in parentheses indicate taxa whose occurrence was uncertain, because their sequences were also similar to that of another taxa or to uncultured cyanobacteria.

Using microscopic analyses, 44 cyanobacterial taxa were detected (18 filamentous and 26 coccoid), 39 of which were classified to the species level, and the remaining five to the genus level. Cyanobacteria in the tested water samples belonged to 20 genera in total (Table 3).

**Table 3:** Comparison of cyanobacterial taxa identified in the microscopic analysis with results from sequencing of excised bands in the DGGE-ITS analysis

**Microscopic analyses**	**DGGE-ITS**
**Coccoid**	**Coccoid**
*Snowella* spp.	
*Woronichinia* spp*.*	
*Chroococcus* spp*.*	
*Aphanothece* spp*.*	
*Microcystis* spp*.*	*Microcystis*
*Aphanocapsa* spp*.*	
*Coelosphaerium* spp*.*	
*Merismopedia* spp*.*	
*Cyanodictyon* spp*.*	
*Lemmermanniella pallida*	
*Rhabdoderma lineare*	
*Synechococcus-*like*/Cyanobium*-like	*Synechococcus*/*Cyanobium (?)*
**Filamentous**	**Filamentous**
*Aphanizomenon* spp*.*	*Aphanizomenon (?)*
*Cuspidothrix issatschenkoi*	*Cuspidothrix issatschenkoi*
*Dolichospermum* spp*.*	*Dolichospermum* spp*.*
*Limnothrix* spp*.*	*Pseudanabaenaceae*
*Planktolyngbya limnetica*	*Pseudanabaenaceae*
*Pseudanabaena* spp*.*	*Pseudanabaenaceae*
*Romeria gracilis*	*Romeria (?)*
*Planktothrix* spp*.*	*Planktothrix*
	*Leptolyngbya*
	*Phormidium (?)*

Question marks in parentheses indicate taxa whose occurrence was uncertain, because their sequences were also similar to that of another taxa or to uncultured cyanobacteria.

**Figure 1:**
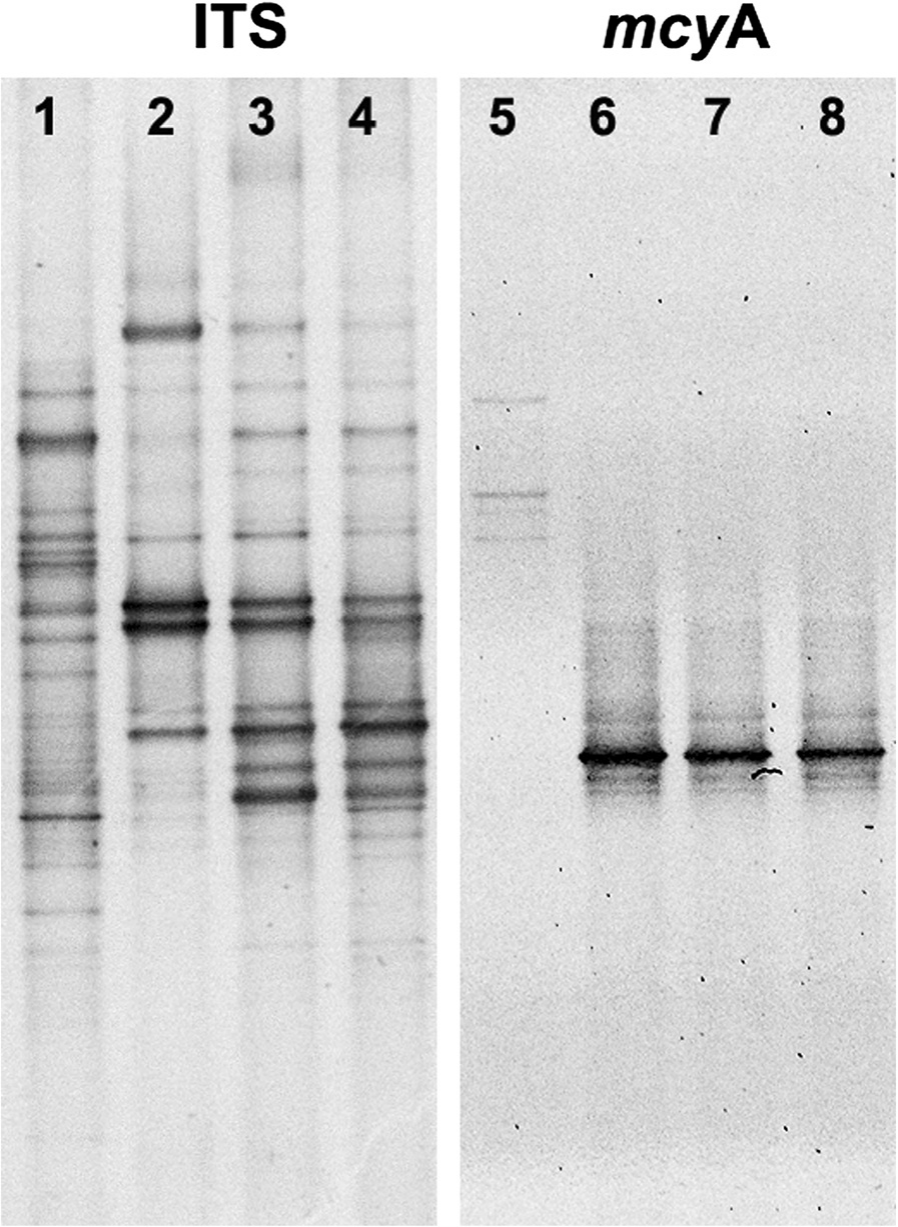
**DGGE profiles of ITS and *mcy*A from lake samples collected in August 2011.** Lane: 1, 5 Lake Mamry; 2, 6 Lake Tałtowisko; 3, 7 Lake Mikołajskie; 4, 8 Lake Bełdany.

During DGGE profiling, 63 different bands were obtained in total for all samples and 41, which is 65% of all bands, were then sequenced and further treated as operational taxonomic units (OTU) (Figure 1). GenBank accession numbers are provided in Table 4. The sequences from obtained OTU were assigned to one species, nine genera and one family (Table 3). Several different sequences could be classified to the same taxon. Seven sequences showed equally high similarity to two different taxa. Eight sequences could not be assigned to any of the taxa whose ITS sequences are present in the GenBank database.

**Table 4:** GenBank accession numbers and similarities to GenBank records of ITS sequences obtained in this study

**Taxa**	**Range of similarity to sequences from the same taxon published in GenBank database**	**Accession numbers**
*Microcystis* spp.	98–99%	KF207563, KF207564, KF207583
*Synechococcus*-like/*Cyanobium*-like	70–99%	KF207565, KF207573, KF207574, KF207576, KF207577, KF207579, KF207580
*Cuspidothrix issatschenkoi*	97%	KF207588
*Dolichospermum* spp. (*Anabaena* spp.)	93–99%	KF207559, KF207560, KF207561, KF207562, KF207589
*Pseudanabaenaceae*	92–96%	KF207566, KF207567, KF207570
*Planktothrix* spp.	98–100%	KF207558, KF207572, KF207591, KF207592
*Leptolyngbya* spp.	86–90%	KF207568, KF207575, KF207586
**Uncertain and unassigned sequences**		
*Aphanizomenon* spp./*Planktothrix* spp.	73–100%	KF207581, KF207582, KF207590
*Phormidium* spp./*Pseudanabaenaceae*	92–94%	KF207571, KF207585
*Romeria* spp.	92%	KF207569
Unassigned		KF207578, KF207579, KF207584, KF207587

The occurrence of almost all filamentous taxa was confirmed by both methods, except for the genera *Leptolyngbya* and *Phormidium*, which were identified by the molecular approach only. However, significant differences existed in the detection of coccoid cyanobacteria using each method. Only the presence of *Microcystis* and picocyanobacteria (*Synechococcus*-like and *Cyanobium*-like groups) were confirmed both microscopically and molecularly. A further 10 coccoid taxa were detected by microscopic analyses (Table 3).

The problems encountered during the analysis of the results from DGGE and sequencing might relate to the small number of sequences for some taxa in the databases. Thus, it is not possible to assign obtained sequences to a specific taxon. Several genera of cyanobacteria found in the microscopic analyses were not represented by any ITS sequence (coccoid - *Lemmermanniella*, *Rhabdoderma*) or partial sequence in GenBank. This partly explains why only a small proportion of the coccoid cyanobacterial taxa identified by microscopy was also detected by molecular analyses. Eight out of 41 sequences obtained by DGGE were not classified to any taxon, which might represent the sequences of the genera mentioned above, and found in the microscopic analysis. These sequences were classified as unassigned (Tables 2 and 4).

The inability to detect some taxa using the molecular method might result from the use of unspecific primers. The ITS primers used in this study are specific for most cyanobacterial taxa found in the microscopic analysis of samples from the studied lakes. For *Limnothrix* and *Pseudanabaena* the CSIF primer has one mismatch within the three bases at the 3′ end (TAC), which are the most specific bases when the sequence is used as the forward primer [9,10]. This might result in a less efficient amplification of such sequences. Indeed, among the tested samples, no sequences could be classified to these two genera, however, there were several sequences indicative of the *Pseudanabaenaceae* family, which includes the genera *Limnothrix* and *Pseudanabaena*.

Differences in the detection of some taxa in both methods can also result from a differential exponential production of amplicons in the PCR. Amplification of various sequences may have different efficiencies. The amount of the DNA template of particular taxon and number of different templates in one sample is important for the efficiency of the PCR reaction [11]. We are not able to estimate what part of the taxonomic diversity can remain undisclosed.

We recorded instances during the study where the obtained sequences were equally similar to two taxa. This occurred seven times during the analyses. These sequences demonstrated the same similarity to *Cyanobium* and *Synechococcus*, *Aphanizomenon* and *Planktothrix*, *Pseudanabaenaceae* and *Phormidium* or to *Romeria* and some uncultured cyanobacteria. Such sequences were classified and included in the Tables 2 and 4 as uncertain. The full list of ITS sequences obtained by DGGE and belonging to various taxa is in Table 4.

Another important consideration when comparing these two methods is the impossibility of sequencing all bands in the gel profile. Very weak bands could not be reamplified and sequenced. The excision of very closely migrating bands was also problematic, because they often did not give homogeneous products after reamplification and DGGE. A total of 35% of the bands from the Mazurian lake samples were not sequenced because of a weak band, problems with reamplification, or presence of more than one sequence in one band. The reason for weak bands, which we could not reamplify and sequence, could be a too low number of cells, as in case of some coccoid taxa (Table 2).

A very important issue in the comparison of these two methods is the cell density at which different taxa are detectable. In the Utermöhl method, it is possible to detect very few cells for various taxa. In this study, we were able to identify cyanobacteria at densities less than 1 cells/mL (for example, *Chroococcus* spp. in Lake Mikołajskie, in September at a density of only 0.1 cells/mL and *Microcystis wesenbergii* in Lake Bełdany, in July at a density of 0.2 cells/mL). We attempted to evaluate a threshold value of cell density, at which it is possible to detect the presence of a given taxon by molecular method. For this, we used two potentially toxic genera, *Microcystis* (coccoid) and *Planktothrix* (filamentous), for which the specificity of the ITS primers has been confirmed [7]. These genera occurred commonly but in varied densities, which made them suit the purpose of the study.

The detection of *Microcystis* using DGGE was possible when the cell density of this genus was greater than 30 cells/mL. Such densities were present in samples from Lake Mamry in July (71 cells/mL), August (34 cells/mL) and September (32 cells/mL). At lower densities, no *Microcystis* was detected by molecular analyses. The highest cell density in which there was no DGGE band corresponding to *Microcystis* was 28 cells/mL (Lake Tałtowisko, August) (Table 5).

**Table 5:** **Comparison of *Microcystis *spp. and *Planktothrix *spp. cell densities evaluated in microscopic analyses and the presence of DGGE-ITS bands corresponding to these taxa in gel profiles**

**Month**	**Lake**	***Microcystis *** **spp.**		***Planktothrix *** **spp.**	
		**Microscope (cells/mL)**	**DGGE-ITS**	**Microscope (cells/mL)**	**DGGE-ITS**
May	Mamry	-	-	62	-
	Tałtowisko	-	-	76	-
	Mikołajskie	-	-	58	+
	Bełdany	-	-	-	+
July	Mamry	71	+	-	-
	Tałtowisko	4	-	4285	+
	Mikołajskie	<1	-	1900	+
	Bełdany	6	-	928	+
August	Mamry	34	+	-	-
	Tałtowisko	28	-	6209	+
	Mikołajskie	7	-	2120	+
	Bełdany	3	-	2660	+
September	Mamry	32	+	-	+
	Tałtowisko	-	-	-	+
	Mikołajskie	-	-	885	+
	Bełdany	4	-	2370	+

For *Planktothrix*, it was not possible to define precisely the minimum detectable cell density for DGGE analysis. Sequences corresponding to *Planktothrix* were found at densities of several hundred or several thousand cells/mL. At densities less than 100 cells/mL, bands corresponding to this taxon either appeared on the gel (Lake Mikołajskie, May) or did not (Lake Mamry, May and Lake Tałtowisko, May). For three samples (Lake Bełdany, May, Lake Mamry, September and Lake Tałtowisko, September), sequences corresponding to the genus *Planktothrix* were identified molecularly, whereas the taxon was not detected by microscopy (Table 5). One of the reasons for the lack of bands in May samples from lakes Mamry and Tałtowisko could be a possible PCR inhibition, although it seemed not to occur in two other samples in the same month. Still, we were not able to set the low limits for *Planktothrix* cells needed to obtain the DGGE bands.

In the gel profiles from DGGE-*mcy*A analyses, a total of 8 different bands were obtained: five of these (63%) were reamplified and sequenced while the other three different bands were too weak to be reamplified (Figure 1). Comparison with GenBank sequences indicated that three obtained sequences belonged to *Microcystis* (similarities to GenBank records: 99%) and two to *Planktothrix* (similarities to GenBank records: 98–99%). (GenBank Accession numbers in GenBank: KF207593–7). Notably, *mcy*A gene sequences corresponding to *Microcystis* spp. were found only in mesotrophic Lake Mamry, whereas in the other, more productive lakes, only *Planktothrix* spp. sequences were found (Table 6). No cyanobacteria possessing the *mcy*A gene were found in the samples collected in May, despite the presence of *Planktothrix* spp. ITS OTUs in two lakes (Table 5). It is difficult to conclude whether this lack of *mcy*A OTUs resulted from the absence of the toxic genes or from too few cells and DNA templates in the samples.

**Table 6:** **Numbers of bands found in DGGE- *mcy *A profiles and taxa identified by comparison of the obtained sequences with the GenBank database**

**Month**	**Lake**	**DGGE- *mcy *** **A**	
		**Bands**	**Taxa**
May	Mamry	0	
	Tałtowisko	0	
	Mikołajskie	0	
	Bełdany	0	
July	Mamry	3	*Microcystis* (2)
	Tałtowisko	4	*Planktothrix* (2)
	Mikołajskie	4	*Planktothrix* (2)
	Bełdany	4	*Planktothrix* (2)
August	Mamry	4	*Microcystis* (3)
	Tałtowisko	4	*Planktothrix* (2)
	Mikołajskie	4	*Planktothrix* (2)
	Bełdany	4	*Planktothrix* (2)
September	Mamry	3	*Microcystis* (2)
	Tałtowisko	1	*Planktothrix*
	Mikołajskie	2	*Planktothrix* (1)
	Bełdany	2	*Planktothrix* (1)

The numbers in parentheses indicate the number of sequences assigned to a given taxon.

## Conclusions

The results from this study demonstrate that microscopic analyses are still indispensable for the identification of the entire taxonomic composition and structure of the cyanobacterial community in a water body and allow the classification of a greater number of taxa with higher accuracy than the molecular method. The results of the DGGE method overlap to a large extent with those of the microscopic analyses, especially in the detection of filamentous cyanobacteria. The attempt to calibrate both methods showed that for *Microcystis* we were able to find a threshold level of cell densities, allowing to detect sequences in DGGE.

The study revealed that there is currently not enough data in public databases to allow the assignment of sequences obtained from environmental samples to coccoid taxa. We suggest that to narrow the gap between these two methods and improve the accuracy of molecular fingerprinting methods in general, more community sequencing as well as isolation of strains from natural environment and sequencing of strains from culture collections should be done.

However, an important advantage of the molecular method already now is the ability to demonstrate the presence of potentially toxic cyanobacteria in a sample, which is not possible using the classical microscopy method. It also allows to identify the diversity of potentially toxic cyanobacteria in a natural environment. Additionally this study provides the optimal conditions for DGGE fingerprinting analyses: the PCR program for (GC)mcyA-Cd1F, mcyA-Cd1R primers; DGGE-ITS and DGGE-*mcy*A designed for samples from freshwater lakes.

## Methods

### Sampling

**Figure 2:**
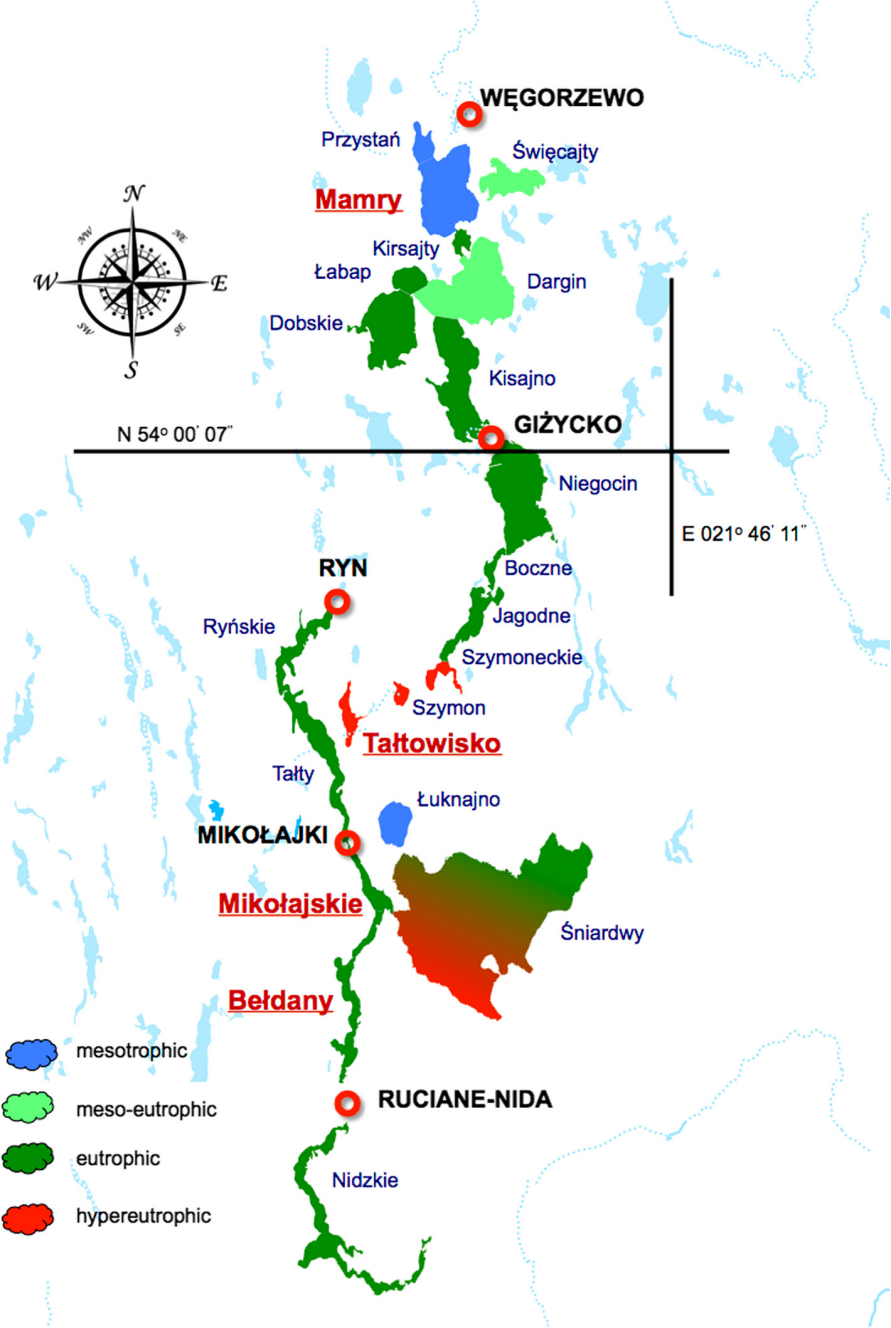
The Great Mazurian Lakes; the names of the studied lakes are marked red and underlined.

Water samples were collected four times during the season in 2011 (May, July, August, September) from the Mamry, Tałtowisko, Mikołajskie and Bełdany lakes. The studied lakes belong to the Great Mazurian Lakes system [12] located in northeastern Poland and represent various trophic status (Figure 2). Integrated water samples, taken at 1.0 m depth intervals from the whole euphotic zone during the mixing period (May) and from the epilimnion during summer thermal stratification (July–September), were stored in plastic containers and transported to the laboratory within 1–5 hours.

Subsamples for microscopic examination were preserved with Lugol′s solution and additionally treated with formaldehyde (final concentration, 0.5%), then stored in a cool dark place until analysis. Samples for microscopic analyses of picocyanobacteria were fixed with formaldehyde only (final concentration, 1%) and also stored in a dark and cool place. Samples for molecular analysis were prepared by filtering 250 mL portions of water through sterile filtration units (Sartorius, 47 mm diameter) through 0.2 μm pore-size polycarbonate filters. Filters were then placed in Eppendorf-type sterile tubes and stored at -20°C until further processing.

### Microscopic analyses

Microscopic analyses were performed according to the Utermöhl method [5]. Phytoplankton was concentrated in 25 or 50 mL sedimentation chambers and examined under an inverted Nikon Eclipse TS 100 microscope with 100×, 200× or 400× magnification. From each sample, at least two subsamples were analysed. If the results of the two subsamples differed significantly from each other, a third subsample was analysed. The final results are mean values calculated from two or three subsample analyses.

To estimate the abundance, biomass and species composition of cyanobacteria, each sample was viewed entirely in terms of identifying less common species. Taxa occurring in greater numbers were measured and counted in 20 randomly selected fields at 200× or 400× magnification. To determine the cyanobacterial taxonomic composition, we used: phytoplankton [13-15] and cyanobacterial keys [16-18], an album of cyanobacterial photographs [19], a series of articles in the journal Fottea (former Czech Phycology) [20-25]. The number of cells in filamentous cyanobacteria was calculated on the basis of a summarised length of cyanobacterial filaments in each sample and the mean length of cells in each identified taxon. The mean cell length was calculated from several-cell measurements of at least 10 specimens from each taxon.

For analyses of picocyanobacteria, between 5 and 10 mL of water sample was filtered through black polycarbonate filters with a pore size 0.2 μm. Picocyanobacterial cells were identified and enumerated under a Nikon Eclipse fluorescent microscope equipped with a 100 W halogen lamp, a colour digital camera (Nikon DXM 1200 F) and CY3 (HYQ) filter (Ex 530-560 nm, DM 570 nm, BA 573-648 nm), [26] with 1.000× magnification.

### Molecular analyses

DNA from frozen filters was isolated using a commercial GeneMATRIX Soil DNA Purification Kit (EURx). PCR reactions were performed using two primer sets (the sequences are given in Table 7) [7,8,27,28].

**Table 7:** Primers used in this study

**Primer**	**Sequence (5′ to 3′)**	**Melting temperatures (°C)**	**Reference**
**ITS**			
(GC)CSIF	G(T/C)CACGCCCGAAGTC(G/A)TTAC	53.8-57.9	[7]
373R	CTAACCACCTGAGCTAAT	45.8	[7,27]
***mcy*** **A**			
(GC)mcyA-Cd1F	AAAATTAAAAGCCGTATCAAA	42.6 (84.1 with CG clamp)	[8,28]
mcyA-Cd1R	AAAAGTGTTTTATTAGCGGCTCAT	50.6	[8,28]

(GC) indicates 5′-CGCCCGCCGCGCCCCGCGCCCGGCCCGCCGCCCCCGCCCC-3′.

The CSIF and 373R primers targeted the end 16S rRNA gene and the fragment of ITS allowed for high-resolution analyses of different cyanobacterial genera. The expected size of the ITS amplicon is 275–325 bp, but in most cases 300 bp. This primer pair allows the amplification of a selected fragment from at least 19 different cyanobacterial taxa including coccoid and filamentous cyanobacteria. Additionally this amplicon mostly gives only one band on the DGGE gel, which makes it especially suitable for this analysis [7].

The mcyA-Cd1F and mcyA-Cd1R primers that targeted the highly conserved condensation domain of *mcy*A gene allowed various cyanobacterial genera of different cell size and organisation of cells, all possessing the *mcy* gene cluster, to be distinguished. This fragment has a small sequence heterogeneity within the genus, which makes it suitable for use in the DGGE method [28]. The expected size of *mcy*A amplicon is 300 bp. A 40-nucleotide GC clamp, which prevents the complete separation of two DNA strands in DGGE, was added to the 5′ ends of the forward primers.

PCR amplification was performed in a Mastercycler epigradient S thermocycler (Eppendorf) in a 25 μL reaction volume containing approximately 20 ng DNA, 0.4 mM of each primer, *Taq* PCR Core Kit (Qiagen) – 0.5 U *Taq* polymerase, 0.2 mM dNTPs, 4 mM MgCl_2_, 1× reaction buffer, 1× Q solution and deionised water.

The temperature cycling conditions for the amplification of the ITS fragment were modified slightly from those of Janse et al. [7]: for the first 20 cycles: preincubation at 94°C for 4 min, the denaturation step lasted 1 min at 94°C, annealing was performed for 1 min with an initial temperature of 62°C, decreasing by 0.5°C after every cycle, to 52°C (to reduce non-specific annealing of the primers), followed by a 1 min elongation step at 72°C. The last 10 cycles were performed at 94°C for 30 s, 51°C for 40 s, and 72°C for 40 s. The temperature cycling was completed with a final step of 10 min at 72°C.

The temperature cycling conditions for the amplification of *mcy*A were as follows: preincubation at 94°C for 4 min, for the first 20 cycles: 94°C for 1 min, with an initial annealing temperature of 59°C, decreasing by 0.6°C each cycle to 47°C for 1 min, 72°C for 1 min; the last 10 cycles – 94°C for 30 s, 47°C for 40 s, 72°C for 40 s and a final step of 10 min at 72°C. In the same study various conditions concerning temperature, time-length of each step and the number of cycles were tested, but the conditions cited above provided the best results.

Similarly as in case of PCR conditions, the DGGE conditions described below were best suited for complex cyanobacterial assemblages analysed in the study. Denaturing gradient gel electrophoresis was carried out in a BioRad ™DCode Universal Mutation Detection System on 1-mm-thick vertical gels, containing 7% (w/v) polyacrylamide (at an acrylamide/bisacrylamide ratio of 37.5:1). The linear gradient of denaturants in the gel increased from 20% to 60% (100% denaturant is defined as 7 M urea and 40% (v/v) formamide). Electrophoresis was performed in TAE buffer at a constant temperature of 60°C for 5 min at 200 V, followed by 16 h at 50 V. Gels were stained for 15 min in a mixture of 14 mL 10,000× SYBR Green (Sigma - Aldrich, Cat No. S9430) and 200 mL deionised water, and subsequently viewed under UV light and photographed. DGGE profiles were analysed with GelCompar II 4.0 software (Applied Maths, Kortrijk, Belgium).

For each sample, a gel profile was obtained. Each band in a single lane, which could be sequenced, corresponded to a single operational taxonomic unit (OTU). Clearly visible, sharp bands were excised from the gel, and if bands existed at the same position in several lanes, at least two of them were excised and sequenced and when they proved to be identical, they were treated as the same OTU. Gel pieces were placed in 40 mL sterile deionised water and incubated at 4°C for 24 h. The eluent was reamplified with the same set of primers and run on DGGE gels to confirm its homogeneity. If the purity of the reamplified samples was confirmed, the PCR products were used as templates for sequencing reactions via the BigDye Terminator Cycle Sequencing Ready Reaction Kit (Applied Biosystems). The sequences were processed using the program 4peaks, version 1.7.1 (Griekspoor and Groothuis, mekentosj.com), and similarity with sequences deposited in GenBank was checked by using the program BLASTn (http://blast.ncbi.nlm.nih.gov/Blast.cgi).

## Abbreviations

DGGE: Denaturing gradient gel electrophoresis; TP: Total phosphorus; TN: Total nitrogen; ITS: Internal transcribed spacer; OTU: Operational taxonomic unit.

## Competing interests

The authors declare that they have no competing interests.

## Authors’ contributions

ABU carried out the molecular analyses, participated in sequence alignment and drafted the manuscript. ABI carried out the microscopic analyses and participated in the molecular analyses. AK participated in the sequence alignment. RJC provided supervision, participated in the design of the study and field sampling, and revised the manuscript. IJ participated in the microscopic analyses, in the design and coordination of the study and helped to draft and participated in revision of the manuscript. All authors read and approved the final manuscript.
